# Structural, dynamic behaviour, in-vitro and computational investigations of Schiff’s bases of 1,3-diphenyl urea derivatives against SARS-CoV-2 spike protein

**DOI:** 10.1038/s41598-024-63345-9

**Published:** 2024-06-01

**Authors:** Saeed Ullah, Atta Ullah, Muhammad Waqas, Sobia Ahsan Halim, Anam Rubbab Pasha, Zahid Shafiq, Suraj N. Mali, Rahul D. Jawarkar, Ajmal Khan, Asaad Khalid, Ashraf N. Abdalla, Hamdy Kashtoh, Ahmed Al-Harrasi

**Affiliations:** 1 Natural and Medical Sciences Research Center, University of Nizwa, Birkat-ul-Mouz 616, Nizwa, Sultanate of Oman; 2 Institute of Chemical Sciences, Bahauddin Zakariya University, Multan, 60800 Pakistan; 3 School of Pharmacy, D.Y. Patil University (Deemed to be University), Sector 7, Nerul, Navi Mumbai, 400706 India; 4 Department of Medicinal Chemistry and Drug Discovery, Dr. Rajendra Gode Institute of Pharmacy, University Mardi Road, Amravati, 444603 India; 5 Substance Abuse and Toxicology Research Center, Jazan University, P.O. Box: 114, 45142 Jazan, Saudi Arabia; 6 Department of Pharmacology and Toxicology, College of Pharmacy, Umm Al-Qura University, 21955 Makkah, Saudi Arabia; 7 Department of Biotechnology, Yeungnam University, Gyeongsan, 38541 Gyeongbuk Republic of Korea

**Keywords:** Schiff’s bases, 1,3-Diphenyl urea, SARS COV-2 spike protein, In silico studies, Docking, MD simulation, Computational biology and bioinformatics, Drug discovery, Infectious diseases

## Abstract

The COVID-19 has had a significant influence on people's lives across the world. The viral genome has undergone numerous unanticipated changes that have given rise to new varieties, raising alarm on a global scale. Bioactive phytochemicals derived from nature and synthetic sources possess lot of potential as pathogenic virus inhibitors. The goal of the recent study is to report new inhibitors of Schiff bases of 1,3-dipheny urea derivatives against SARS COV-2 spike protein through in-vitro and in-silico approach. Total 14 compounds were evaluated, surprisingly, all the compounds showed strong inhibition with inhibitory values between 79.60% and 96.00% inhibition. Here, compounds **3a** (96.00%), **3d** (89.60%), **3e** (84.30%), **3f** (86.20%), **3g** (88.30%), **3h** (86.80%), **3k** (82.10%), **3l** (90.10%), **3m** (93.49%), **3n** (85.64%), and **3o** (81.79%) exhibited high inhibitory potential against SARS COV-2 spike protein. While **3c** also showed significant inhibitory potential with 79.60% inhibition. The molecular docking of these compounds revealed excellent fitting of molecules in the spike protein receptor binding domain (RBD) with good interactions with the key residues of RBD and docking scores ranging from − 4.73 to − 5.60 kcal/mol. Furthermore, molecular dynamics simulation for 150 ns indicated a strong stability of a complex 3a:6MOJ. These findings obtained from the in-vitro and in-silico study reflect higher potency of the Schiff bases of 1,3-diphenyl urea derivatives. Furthermore, also highlight their medicinal importance for the treatment of SARS COV-2 infection. Therefore, these small molecules could be a possible drug candidate.

## Introduction

SARS-CoV-2 is a single-stranded RNA (ssRNA) virus that is present in capsule form^1^. The ssRNA of Coronaviruses is typically 26–32 kilobases long, segment-free, and covered in a lipid sheath with average size between 80 and 120 nm, while its diameters are mostly in the range of 50 nm to 200 nm, with ~ 40,000 kDa molecular weight. The subfamilies *Torovirinae* and *Coronavirinae* of the family *Coronaviridae* serves as reservoirs and vectors for coronaviruses, according to ICTV (International Committee on Taxonomy of Viruses). Examples of the beta coronavirus genus, which is a member of the Coronavirinae subfamily, include the viruses MERS-CoV and SARS-CoV. This virus family includes the novel SARS-CoV-2, which is known as COVID-19 because of its emergence in late 2019. Four structural proteins including spike, membrane, envelope, and nucleocapsid proteins constitutes the structure of SARS-CoV-2. The term "coronavirus" is well named since the virus's spike proteins protrude outward from its exterior, giving it a crown-like appearance. Spike proteins are linked to specific receptors, such as the human angiotensin-converting enzyme 2 (hACE2) receptor found in human lung cells that are essential for the attachment of viruses to the host^2–5^. Envelope proteins are necessary for viral particle synthesis and release, while membrane proteins are crucial for viral assembly and are responsible for maintaining the structure of the envelope. The nucleocapsid, which is made up of nucleocapsid proteins, contains the viral genome. Virus packing and RNA replication both require these proteins.

The distinctive spikes comprising 74 spike proteins with 20 nm length in a trimeric form that provide a halo effect on the coronaviruses make them easy to spot^5,6^. Two functional subunits, S1 and S2, make up the Spike protein receptor binding domain (RBD)^7^. The spike protein's exposed region S1 interacts with the hACE2 receptor and helps the virus enter host cells by undergoing conformational changes^8^. The core portion of the S2 has transmembrane and fusion peptide domains. The host cell's membrane can fuse with the virus more easily that is why the fusion peptide is extremely crucial in drug target^9^. When the Spike protein binds to the receptor, a type 2 transmembrane (TM) protease serine 2 (TMPRSS2) located on the host cell membrane activates the Spike protein and facilitates viral entrance into the cell^10^. The fusion causes the viral genome to enter the host cell, where it replicates its RNA to start the infection^11^. The searching of new drugs which can successfully suppress the spike protein of SARS-CoV-2 is still going on^12^. As of right now, no drug has been approved to treat the coronavirus illness. Still, to assess coronavirus treatments, clinical trials of synthetic pharmacological molecules have underway^13^. Mostly synthetics drugs are in clinical trials which are inhibiting the viral protein^14^. Overcoming the issue of SARS-CoV-2 genome change and viral resistance to medicines and vaccines over time remains a challenge^15–18^. Also, vaccines have been developed as an effective agents but still possess some modest side effects^19–21^. Viruses can mutate over time, giving rise to novel strains that are resistant to current treatments and vaccinations and capable of completely or partially preventing immunisation. The development of small new inhibitors could provide treatment alternatives that effectively counteract emerging variants. Designing inhibitors against the spike protein which fit well into the key parts of the spike protein could aim for a broad-spectrum effect, targeting multiple variants or even different coronaviruses. Since drugs developed to treat SARS-CoV-2 could provide an effective first line of defence against future coronaviruses, their development is more rational and preferable^22^. The use of in-vitro and in silico techniques, which entail the potency and the favourable interactions of the lead molecules, has become more important in the process of finding new drugs. These approaches can prevent the virus from spreading with fewer adverse effects at a lower cost^23,24^. Patients with SARS-CoV-2 illness still require urgent complementary and alternative therapies, and experiences with herbal and synthetic therapy are unquestionably worthwhile researching. In this context we designed current studies to explore the inhibitory activity of the Schiff bases of 1,3-dipheny urea against SARS-CoV-2 spike protein as these compounds are already reported for their medicinal importance via targeting the key catabolic enzyme of carbohydrates (α-glucosidase)^25^.

**Figure 1:**
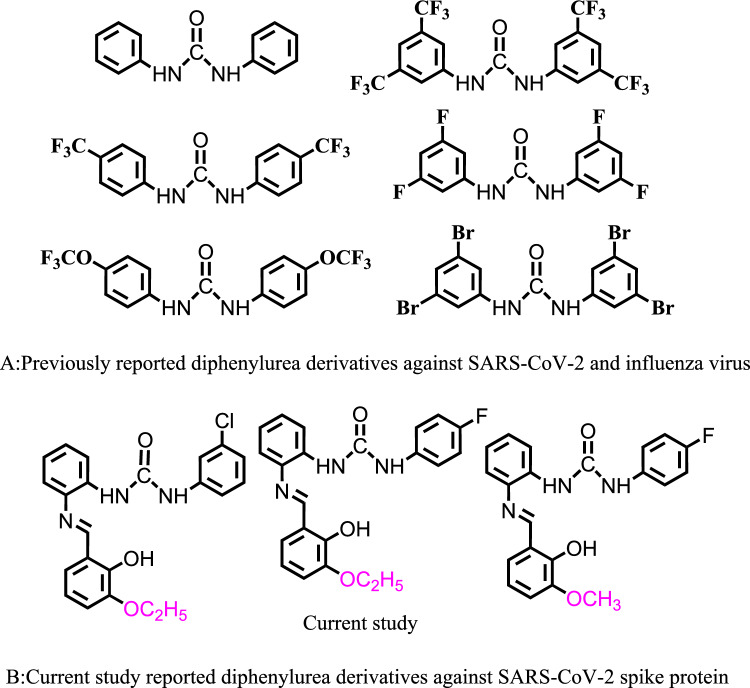
(**A**) Previously reported and (**B**) current reported diphenylurea derivatives against SARS-CoV-2 and influenza virus.

Several medicines and bioactive substances with a wide range of varied medicinal and pharmacological activities are framed inside the favored structures that make up urea. Various compounds with urea motifs are authorized as marketing medications by food and drug administration (FDA). They do indeed represent promising therapeutic prospects and enjoy a prominent position in academic research as well as synthetic and medicinal chemistry^26^. Due to the presence of an electron pair in the sp2 hybridized orbital of the imine, Schiff bases are substances of significant biological importance that contain an azomethine or imine linkage. These compounds exhibit remarkable pharmacological properties like antitumor, anti-fungal, antiviral, and antibacterial activities^27,28^. Particularly intriguing as potential anticancer medication candidates are Schiff bases^29–31^. In addition, urea derivatives are well-known anti-diabetic medications that work by inhibiting the α-glucosidase enzyme. Several inhibitors for this class have already been found, demonstrating their potential for application in drug discovery^32–35^. Furthermore, Schiff bases of 1,3-dipheny urea are also reported for Alzheimer disease and as for their antioxidant and anti-inflammatory potential and anti-epoxide hydrolase which is a significant advantage to use against SARS-CoV-2 infection^36–39^. Apart from this Schiff bases are reported for their anti-acetyl cholinesterase^40^ Moreover, diphenylurea derivatives are reported *in*-*vitro* and in vivo inhibitors for SARS-CoV-2 and influenza virus and their action of mechanism is also reported that block their cellular entry (Fig. 1)^41,42^. We were interested in finding novel compounds against SARS-CoV-2, especially for spike protein^43,44^. These reported findings of Schiff bases of 1,3-dipheny urea directed us to evaluate the inhibitory capability of Schiff bases of 1,3-dipheny urea against SARS-CoV-2 via specific inhibition of its spike protein. We reported these findings for the first time to inhibit SARS-CoV-2 spike protein.

## Results and discussion

### In vitro inhibition of SARS-CoV-2 spike protein

**Figure 2:**
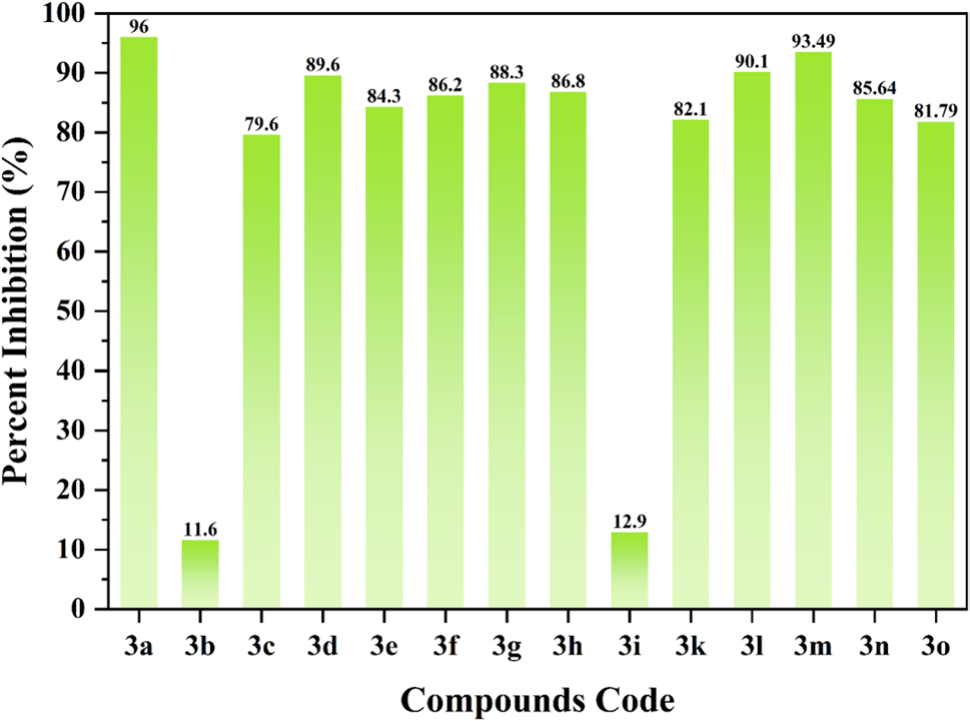
Graphical representation of compounds **3a**–**3i** and **3k**–**3o** against SARS-CoV-2 Spike protein.

**Figure 3:**
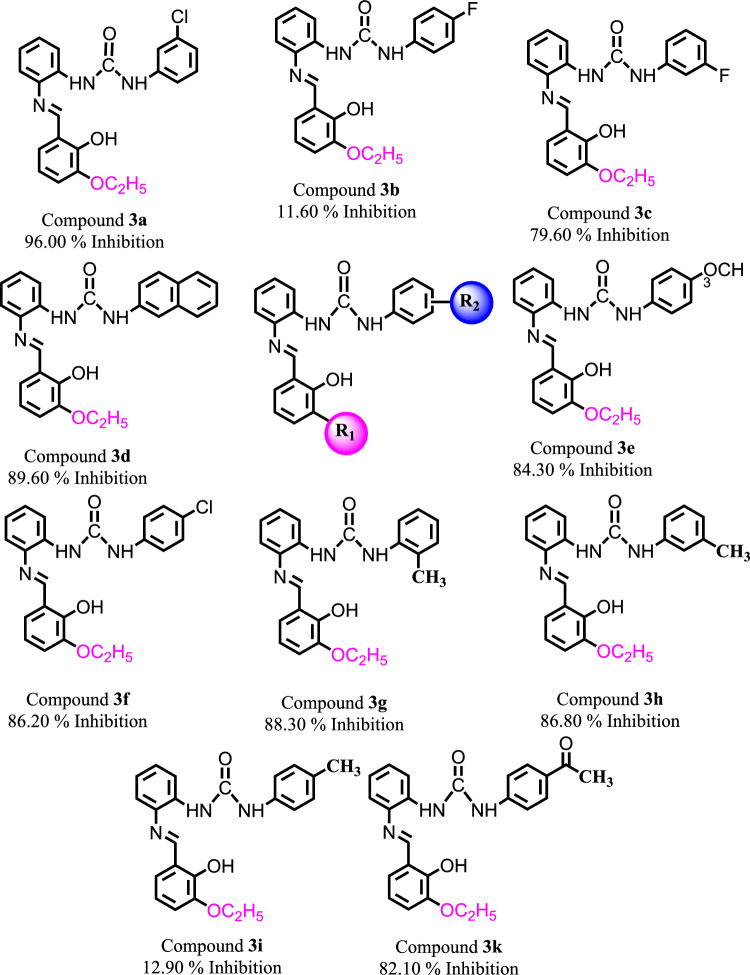
Chemical structures of newly identified inhibitors (**3a**–**3k**) of Spike protein.

In the current study, therapeutic importance of fourteen compounds (**3a**–**3o**) was evaluated for the treatment of SARS-CoV-2 by targeting its Spike protein (Fig. 2). Fortunately, we have identified new spike protein inhibitors with high to significant inhibitory potency (Fig. 3). Except compounds **3b**, and **3i**, the rest of the synthetic scaffolds are active in the range of 79.60–96.00% inhibition. These evaluated compounds were categorized into two groups, i.e., group 1 and 2. In group 1, there are total 11 compounds having similar R_1_-OC_2_H_5_ group with diverse R_2_ substituents. This indicates that different R_2_ substituents in group 1 compounds might be responsible for the difference in their activity against SARS-CoV-2 spike protein.

For instance, compound **3a** with the *meta*-chlorine substitution exhibited the highest inhibitory potential in the series with the highest 96.00% inhibition, on the other hand, compound **3b** with *para*-fluorine substitution is inactive. However, when the fluorine substituent position was changed in compound **3c** at *para*-fluorine, **3c** displayed significant inhibitory capability i.e., 79.60% inhibition. In compound **3d,** substitution of naphthyl increased the inhibitory capability of **3d** with 89.60% inhibition as compared to **3c**. Likewise, compound **3e**, with *para*-methoxy substitution also displayed high inhibitory potential against spike protein with 84.30% inhibition. Moreover, compound **3f** with *para*-Cl substitution also displayed high inhibitory activity with 86.20% inhibition. The effect of methyl group was evaluated in compounds **3g**–**3i** which exhibited almost similar inhibitory capability. We observed that **3g** with *ortho*-CH_3_ and **3h** with *meta*-CH_3_ substituents also exhibited high inhibitory potential with 86.30%, and 86.80% inhibition, respectively. While compound **3i** with *para*-CH_3_ exhibited < 50% inhibition and is declared as inactive against SARS-CoV-2 spike protein. Compound **3k** with *para*-COCH_3_ also resulted into high inhibitory activity with 82.10% inhibition.

**Figure 4:**
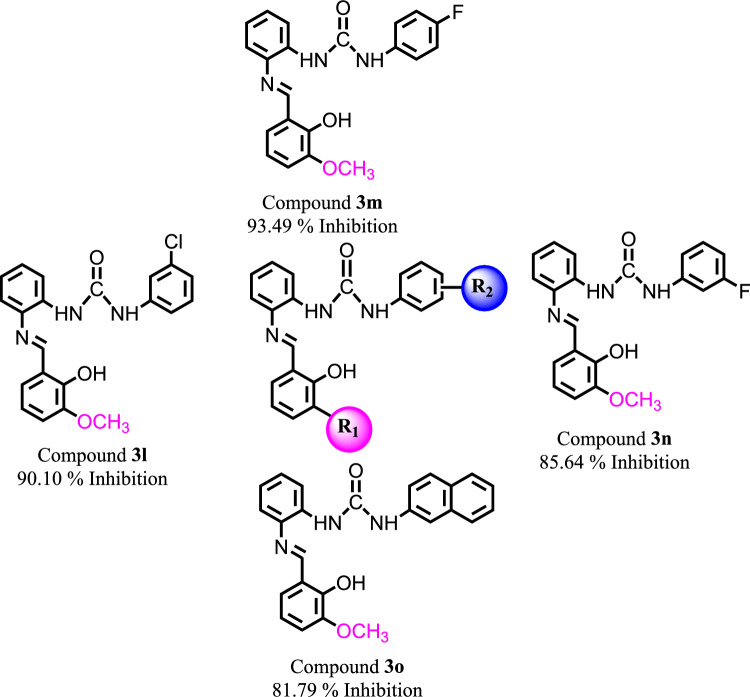
Chemical structures of Group 2 compounds (**3l**–**3o**) which displayed excellent inhibition of Spike protein.

Group 2 comprises of four compounds with similar R_1_-OCH_3_ and with different R_2_ substitutions. In group 2, all the compounds exhibited higher inhibitory capability against SARS-CoV-2 spike protein (Fig. 4). Compound **3l** with *meta*-chlorine substituent resulted into excellent inhibitory potential with 90.10% inhibition. In compounds **3m** and **3n**, the effect of fluorine was evaluated for their inhibitory potential against SARS-CoV-2 spike protein. Compound **3m** with *para*-fluorine exhibited higher inhibitory potential with 93.49% inhibition, as compared to compound **3n** with *meta*-fluorine and 85.64% inhibition. Whereas compound **3o** with naphthyl substitution also demonstrated good inhibitory potency with 81.79% inhibition.

### Molecular docking of chosen compounds

**Figure 5:**
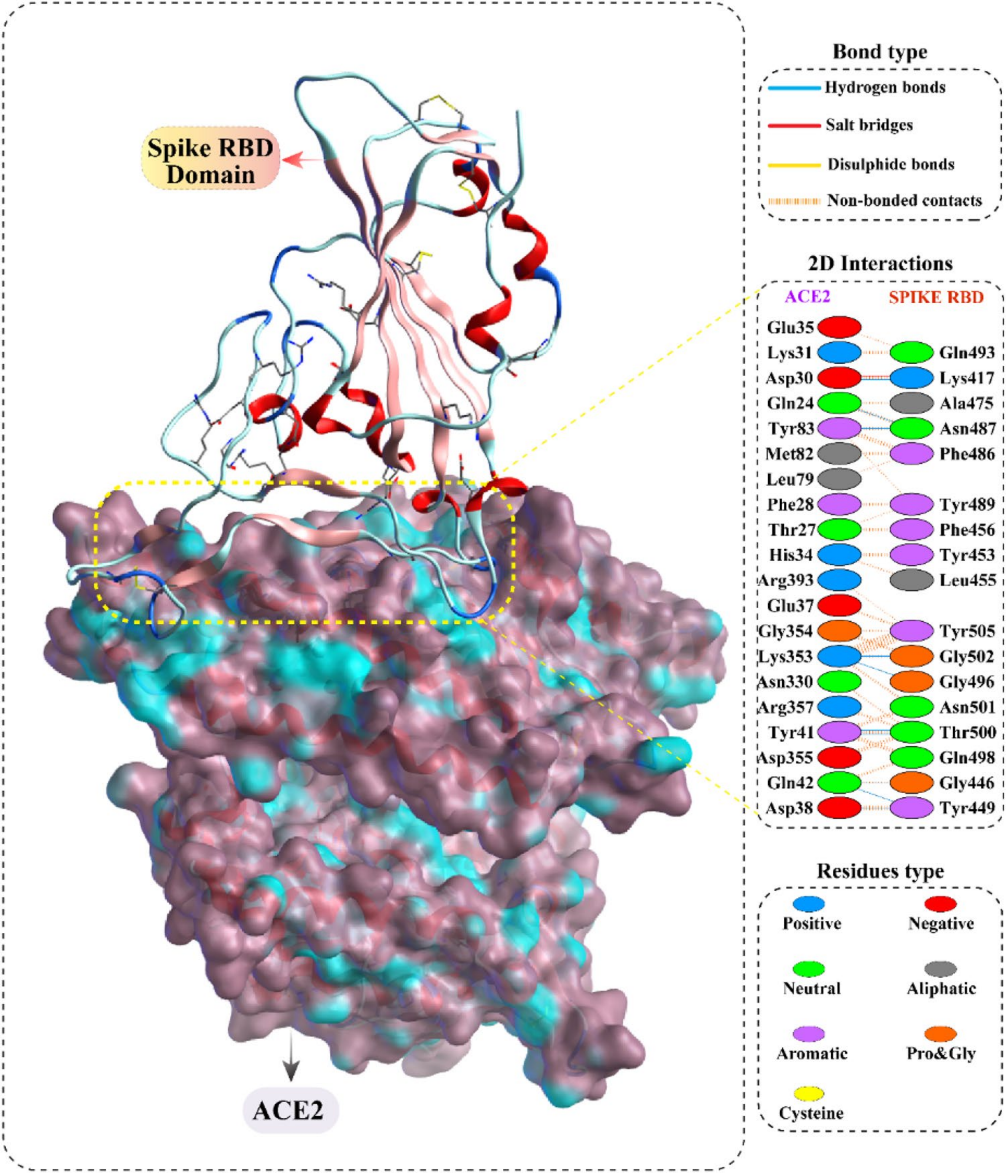
The receptor binding domain (RBD) of SARS-CoV-2 spike protein is shown in complex with the human ACE2 receptor. The residual interactions at this protein–protein interface are reported in the box.

For docking analysis, we used Spike protein RBD in complex with hACE2 receptor. Several residues of hACE2 receptor including GLN24, ASP30, ASP38, TYR41, TYR41, GLN42, GLN42, TYR83, LYS353, LYS353 and ASP30 interacts with RBD of Spike protein (residues: ASN487, LYS417, TYR449, THR500, THR500, GLY446, TYR449, ASN487, GLY502, GLY496, and LYS417) to stabilize Protein–Protein interaction mainly through hydrogen bonds and salt bridges. The detailed interaction of the spike protein and hACE2 receptor is given in (Fig. 5).

**Figure 6:**
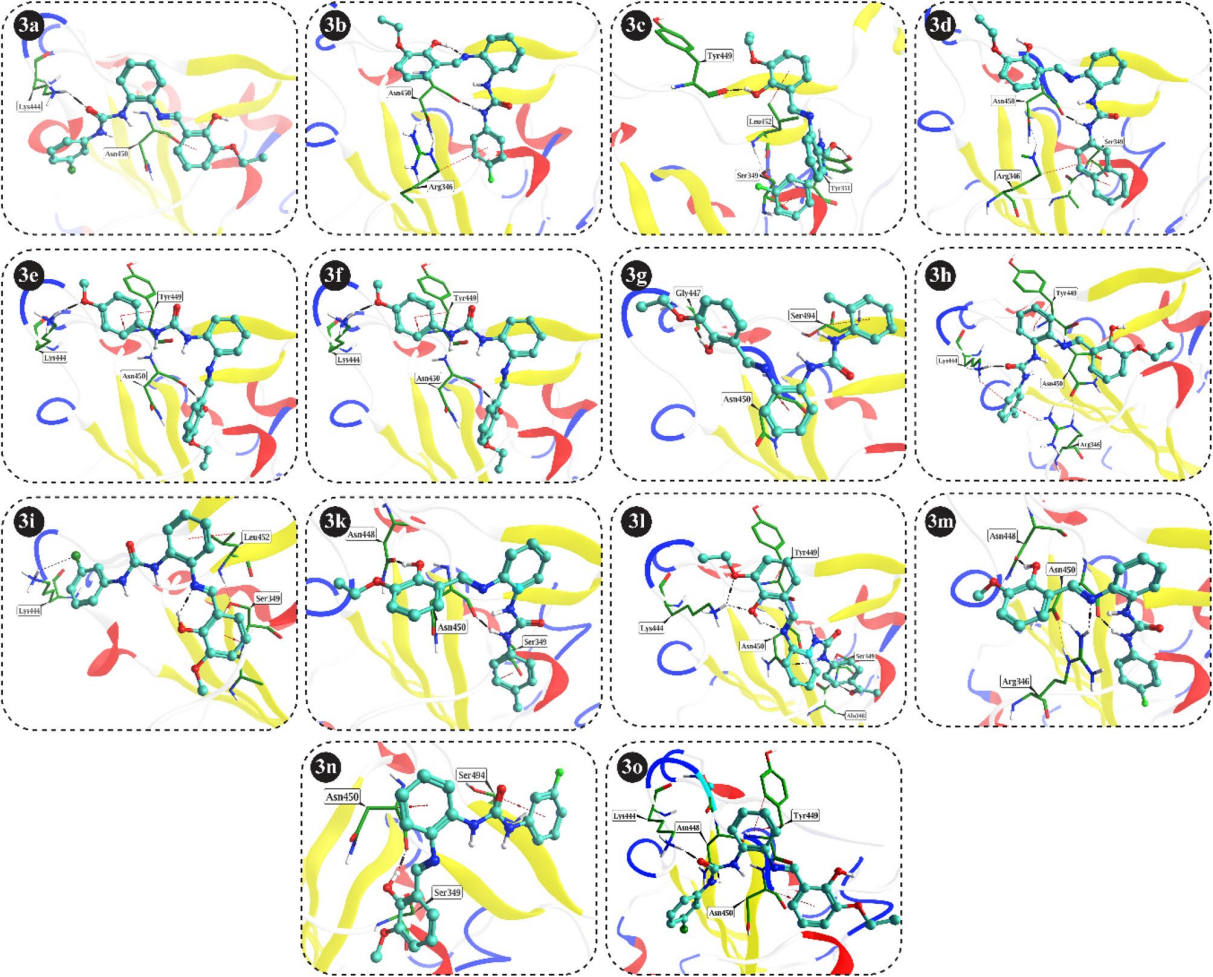
The interactions of compounds **3a**–**3o** are shown at the RBD of spike protein. Hydrogen bonds and hydrophobic interactions are illustrated black and red dotted lines, respectively.

The software Molecular operating environment (MOE) version (2022.09) was used to analyse the docking interaction of inhibitors. The human ACE2 receptor (hACE2) binds to the spike protein at its receptor binding domain (RBD). Therefore, RBD was selected as the site at which the chemical compounds were docked. The protein–ligand interaction is stabilized by the Hydrogen bonds (H-bond) and π–hydrogen (π–H) bonds between the compounds (**3a–3o**) and the RBD of spike protein. Compounds **3l, 3a** and **3o** showed the highest docking score of − 5.60 kcal/mol, − 6.69 kcal/mol and − 5.50 kcal/mol followed by **3g, 3e, 3k, 3n, 3f, 3d, 3i, 3m, 3c, 3h,** and **3b** (Table 1). Furthermore, compound **3a** formed two hydrogen bonds and two π–H bonds with ASN448, LYS444, TYR449 and ASN450, while **3b** mediates contacts with ASN450, ARG346 through hydrogen and π–H bonds. The inhibitor **3c** established a hydrogen bond with residue TYR449 and TYR351 and two π-H bonds with SER349, and LEU452. The **3d** make a stable complex with spike protein by mediating H-bond with ASN450 and two π-H bonds with ARG346 and SER349. Likewise, **3e** creates two hydrogen bonds and a π-H bond with ASN450, LYS444 and TYR449, while **3f** also forms two hydrogen bonds and a π–H interaction with ASN450, SER349, and ALA348. The compound **3g** makes a H-bond and π–H bond with GLY447 and SER494, and compound **3h** makes two H-bonds with ASN448 and LYS444) and two π–H bonds with ARG346 and LYS444, likewise, LYS444 and SER349 stabilizes **3i** by mediating H-bonds and π–H at RBD of Spike protein. Similarly, the compound **3k** make two H-bond and π–H bonds with ASN450, ASN448 and SER349. The compound **3l** make a constant complex by mediating multiple hydrogen bonds and one π–H bond with ASN450, LYS444, SER349, LYS444 and TYR449. The compound **3m** makes contacts with ASN450, ASN448 and ARG346 through H-bonds, while **3n** interacts with ASN450 (2x) through H-bond and π–H bond. Moreover, compound **3o** is stabilized through hydrogen and π–H bonds by LYS444 and TYR449. This data supports robust molecular interactions of inhibitors at RBD of spike protein. The interactions of compounds **3a–3o** are illustrated in (Fig. 6). The docking scores of these molecules are highly negative (tabulated in Table 1) reflecting excellent binding affinities of these compounds for RBD of spike protein.

**Table 1:** Docking results compounds **3a–3o** with RBD of Spike protein.

Compounds	Docking score (kcal/mol)	Ligand atom	Residues of RBD	Interaction type	Distance (Å)	Per interaction energy (kcal/mol)
**3a**	− 6.69	N5	ASN448	HBD	2.88	− 2.6
		O2	LYS444	HBA	2.88	− 9.0
		6-ring	TYR449	H–π	3.30	− 0.9
		6-ring	ASN450	H–π	3.65	− 0.7
**3b**	− 4.74	N5	ASN450	HBD	2.74	− 5.4
		6-ring	ARG346	H–π	3.55	− 0.5
**3c**	− 4.91	O39	TYR449	HBD	2.61	− 3.1
		O2	TYR351	HBA	2.57	− 3.1
		6-ring	SER349	H–π	3.30	− 0.8
		6-ring	LEU452	H–π	3.62	− 0.6
**3d**	− 5.23	N5	ASN450	HBD	2.69	− 3.9
		6-ring	ARG346	H–π	3.69	− 0.5
		6-ring	SER349	H–π	3.45	− 0.5
**3e**	− 5.39	O39	ASN450	HBD	2.55	− 3.6
		O49	LYS444	HBA	2.83	− 5.2
		6-ring	TYR449	H–π	3.18	− 1.5
**3f**	− 5.23	N5	ASN450	HBD	2.81	− 2.7
		O2	SER349	HBA	2.75	− 2.1
		6-ring	ALA348	H–π	3.77	− 0.5
**3g**	− 5.48	O39	GLY447	HBD	2.85	− 3.5
		6-ring	SER494	H–π	3.03	− 0.5
**3h**	− 4.94	N5	ASN448	HBD	2.86	− 2.8
		O2	LYS444	HBA	2.54	− 9.3
		6-ring	ARG346	H–π	3.48	− 1.1
		6-ring	LYS444	H–π	3.29	− 0.5
**3i**	− 5.13	CL28	LYS444	HBA	3.85	− 0.5
		6-ring	SER349	H–π	3.40	− 0.6
**3k**	− 5.31	N5	ASN450	HBD	2.69	− 3.5
		O39	ASN448	HBD	2.65	− 3.9
		6-ring	SER349	H–π	3.50	− 0.5
**3l**	− 5.60	N5	ASN450	HBD	3.12	− 2.1
		O39	LYS444	HBA	2.66	− 1.0
		O42	SER349	HBA	2.74	− 3.5
		O47	LYS444	HBA	2.97	− 2.0
		6-ring	TYR449	H–π	3.24	− 0.9
**3m**	− 5.08	N5	ASN450	HBD	2.67	− 3.2
		O39	ASN448	HBD	2.63	− 3.0
		N27	ARG346	HBA	2.78	− 2.5
**3n**	− 5.28	O39	ASN450	HBA	2.58	− 2.8
		6-ring	ASN450	H–π	3.27	− 0.6
**3o**	− 5.50	O2	LYS444	HBA	2.88	− 5.4
		6-ring	TYR449	H–π	3.54	− 0.7

*HBA* hydrogen bond acceptor, *HBD* hydrogen bond donors, *H–π* (pi-proton interaction belongs to hydrophobic interaction).

### BOILED-egg prediction (GI absorption and brain penetration)

We conducted an analysis on a set of recently created compounds labelled as **3a** to **3o**. This analysis was performed utilizing the 'BOILED-egg model' through the 'SwissADME' webserver, accessible at http://www.swissadme.ch/index.php. This model offers a visually intuitive and efficient way to assess the polarity and lipophilicity of these chemical compounds. It’s utility lies in its ability to aid drug development by streamlining the screening of chemical libraries. The BOILED-Egg model offers a swift, easily reproducible, statistically sound approach for predicting passive absorption in the gastrointestinal tract and the potential of small molecules to cross the blood–brain barrier—an essential aspect of drug discovery and development.

**Figure 7:**
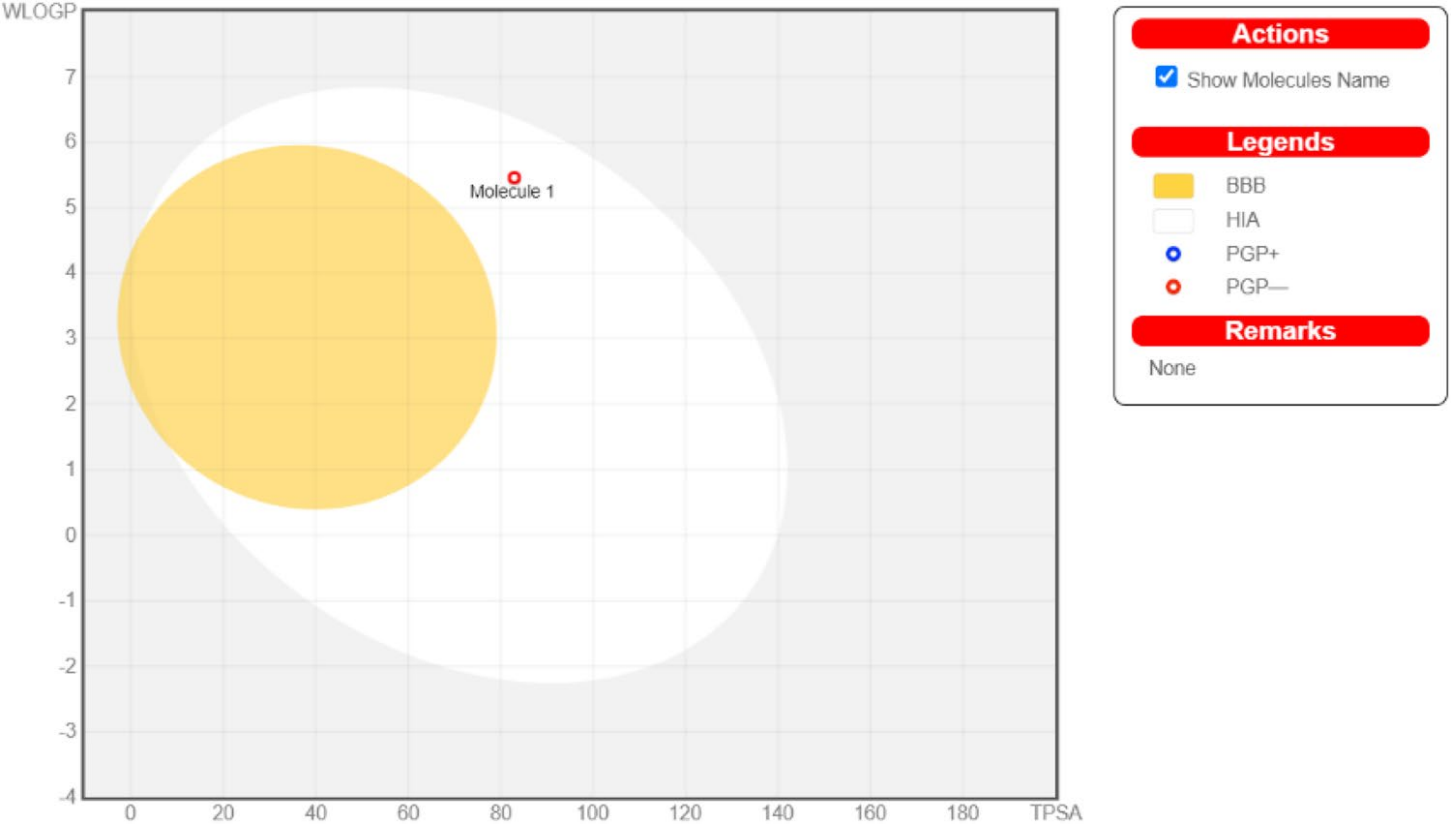
BOILED-egg model analysis for compound **3a**.

In this model, the white region indicates a high likelihood of passive absorption in the gastrointestinal tract, while the yellow area (resembling the yolk) suggests a strong probability of successfully crossing the blood–brain barrier. Furthermore, the blue color of the compound indicates active efflux by P-glycoprotein, labelled as (PGP^+^), whereas the red color signifies that P-glycoprotein is not actively facilitating efflux, indicated by (PGP^-^). The analysis revealed that all the compounds displayed negative P-glycoprotein profiles and were situated within the white zone of the boiled-egg model (Fig. 7).

### Predictive ADME (absorption, distribution, metabolism, excretion) studies

In the current research, we conducted an evaluation of the ADME (Absorption, Distribution, Metabolism, and Excretion) characteristics of a series of synthesized compounds, specifically compounds **3a** through **3o**. To carry out this assessment, we utilized the 'QikProp' module, developed by Schrodinger, Inc. in New York, USA, 2023. Table 2 presents a comprehensive summary of the computed ADME properties, encompassing ten different descriptors: #stars (highlighting properties that deviate from the optimal range for 95% of drugs), volume, QPlogPo/w (predicting the octanol/water partition coefficient), QPlogHERG (predicting interaction with the Human ether-a-go-go-related gene), QPPCaco (predicting apparent Caco-2 cell membrane permeability in nm/s), QPPMDCK (predicting apparent Madin-Darby canine kidney (MDCK) cell permeability in nm/s), #metab (indicating the number of potential metabolic reactions), % Human Oral Absorption (HOA), PSA (polar surface area), and RO5 (compliance with Lipinski's Rule of Five).

**Table 2:** ADME results of compounds **3a** to **3o**.

Comp	#Stars	Volume	QPlogPo/w	QPlogHERG	QPPCaco	QPPMDCK	#metab	% human oral absorption	PSA	RO5
**3a**	**1**	**1308.319**	**4.745**	**− 6.185**	**976.024**	**1727.443**	**4**	**100**	**80.98**	**0**
**3b**	1	1278.327	4.38	− 6.212	737.04	951.297	3	100	82.848	0
**3c**	1	1280.258	4.488	− 6.164	976.954	1266.585	4	100	81.019	0
**3d**	3	1404.071	5.193	− 6.941	929.787	667.093	3	100	81.104	1
**3e**	1	1349.153	4.255	− 6.352	585.578	409.003	4	100	90.995	0
**3f**	1	1305.935	4.634	− 6.218	737.811	1298.694	3	100	82.844	0
**3g**	0	1315.38	4.489	− 6.059	914.306	660.904	5	100	81.205	0
**3h**	0	1324.385	4.562	− 6.196	972.198	699.342	5	100	81.059	0
**3i**	0	1322.344	4.452	− 6.228	736.16	524.473	4	100	82.811	0
**3k**	1	1396.88	3.68	− 6.412	186.839	119.203	3	89.146	111.722	0
**3l**	0	1242.625	4.19	− 6.031	622.111	1067.851	4	100	83.137	0
**3m**	1	1212.815	3.936	− 5.944	643.133	818.054	3	100	83.121	0
**3n**	1	1216.325	3.94	− 6.037	611.655	771.149	4	100	83.203	0
**3o**	2	1336.056	4.637	− 6.721	625.391	436.243	3	100	83.073	0

Significant values are in bold.

#stars : Number of property or descriptor values that fall outside the 95% range of similar values for known drugs; volume: Total solvent-accessible volume in Å^3^ using a probe with a 1.4 Å radius; QPlogPo/w: Predicted octanol/water partition coefficient (< 5); QPlogHERG: Predicted IC_50_ value for blockage of HERG-K^+^ channels; QPPCaco: Predicted apparent Caco-2 cell permeability in nm/sec (< 25 poor, > 500 great); QPPMDCK: Predicted apparent MDCK cell permeability in nm/sec; #metabol: the number of likely metabolic reactions; PSA: Polar surface area; RO5: Lipinski rule of 5 helps in distinguishing between drug like and non-drug like molecules. % Human Oral Absorption (< 25% poor, > 80% high).

The examination of the #stars values across all compounds revealed a consistent range of 1 to 3, signifying generally favourable ADME properties. Most of the compounds exhibited moderate lipophilicity, as indicated by the QPlogPo/w values, which fell within the range of 3.68 to 5.193. Caco-2 cell permeability, an *in-vitro* model assessing a compound's ability to traverse the intestinal barrier and influence oral drug absorption, provided promising results. All compounds in the series (**3a** to **3o**) demonstrated Caco-2 cell permeabilities exceeding 500 nm/s (for example, **3a** exhibited a permeability of 976.024 nm/s). This suggests that these compounds have a high likelihood of being absorbed into the bloodstream, potentially enhancing their efficacy as oral medications. However, a noteworthy concern emerged as several of the compounds showed potential for blocking HERG-K^+^ channels, which could lead to cardiotoxicity and arrhythmia. This highlights the need for further optimization to address this issue. Regarding metabolic reactions, the set of molecules displayed a range of 1 to 5 reactions, with **3a**, for instance, undergoing 4 metabolic reactions. All analogues demonstrated nearly 100% human oral absorption (% HOA), indicating a favourable potential for oral bioavailability, except for compound **3k**, which exhibited an absorption rate of 89.146%. Additionally, the majority of compounds adhered to good drug-likeness characteristics, with no more than one violation of Lipinski's Rule of Five (RO5), except for **3d**. This suggests that most of the synthesized compounds possess properties conducive to drug development and warrant further investigation as promising candidates. Other properties such as volume, QPPMDCK (**3a**: 1727.443), and PSA (**3a**: 80.98) were also within their acceptable ranges, as outlined in Table 2.

### Molecular dynamics analysis

**Figure 8:**
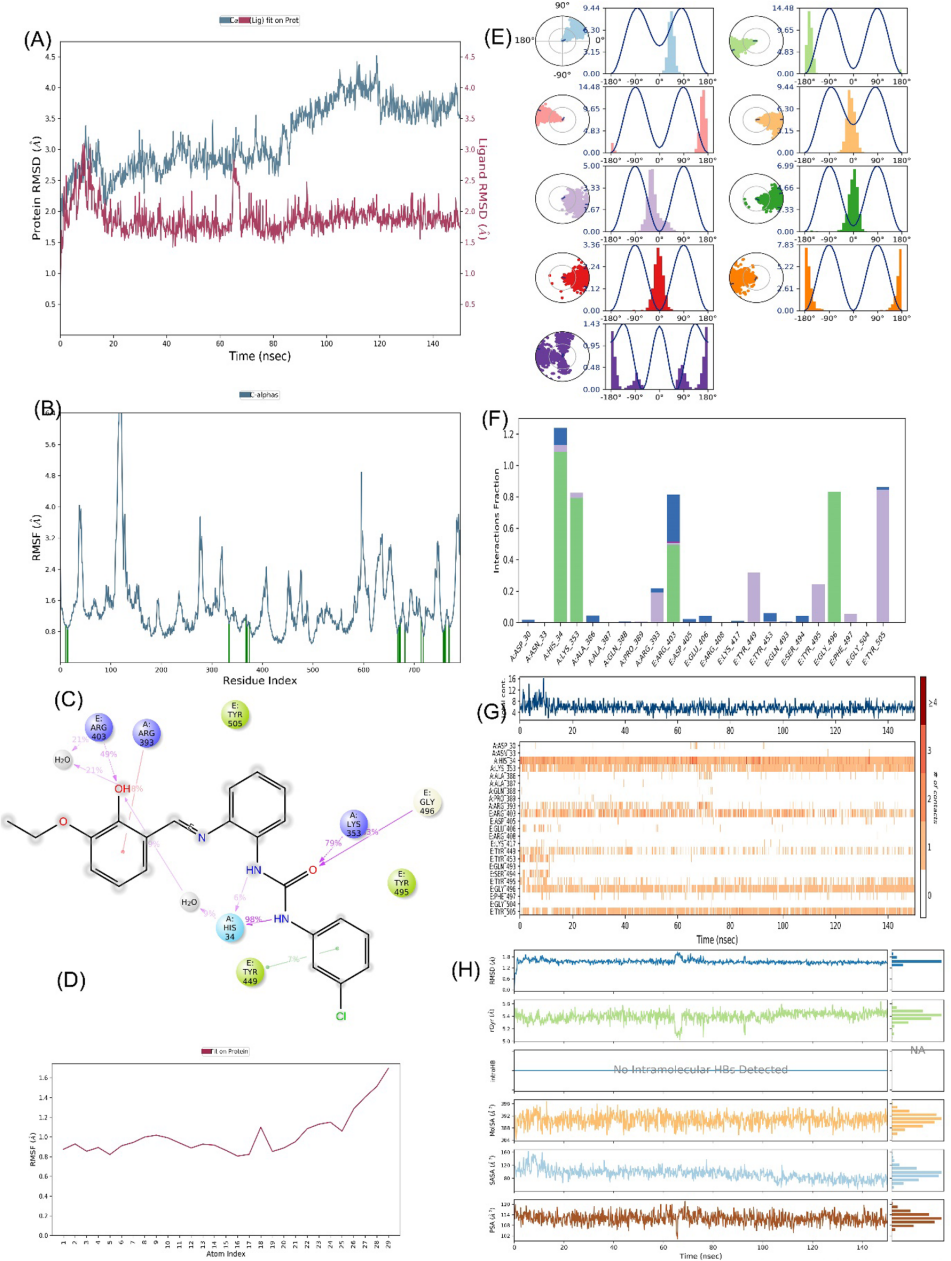
MD simulation analysis for complex **6moj_3a** (**A**) RMSD plot; (**B**) RMSF analysis; (**C**) Ligand–Protein contact plot; (**D**) Ligand RMSF; (**E**) Ligand torsion profile; (**F**) Protein–Ligand interaction plot; (**G**) a timeline representation plot representing such interactions with amino acid residues over the simulation period of 150 ns and (**H**) Ligand properties for compound **3a**.

We conducted a comprehensive assessment of the stability of the top-docked ligand, denoted as 3a_6MOJ, through an extensive 150 ns (ns) molecular dynamics simulation (MD) employing the 'Desmond' software developed by Schrodinger, LLC, New York, 2023. The MD system encompassed a total of 73,005 atoms, which included 73,005 water molecules. In this analysis, a total of 792 residues with 12,516 atoms were involved. During these dynamic simulations, we meticulously evaluated the average atomic displacements within specific time intervals, employing the 'RMSD' (Root Mean Square Deviation) parameter. RMSD analysis consistently indicated a stable conformation. When ligand 3a was bound to the target 6MOJ, the Cα-RMSD backbone values remained consistently below 4.0 Å, and the 'Lig_fit_Prot' values consistently stayed below 3.0 Å (refer to Fig. 8A). This affirmed the overall stability of the entire complex over the duration of 0–150 ns. Furthermore, we scrutinized local variations in the protein chains using the 'RMSF' (Root Mean Square Fluctuation) plot (Fig. 8B). Although we observed minor fluctuations in selected proteins, no significant changes transpired, underscoring the anticipated flexibility of these residues. In addition to RMSD and RMSF analyses of ligand (Fig. 8C–E), we delved into the 'protein–ligand' interactions (Fig. 8F), unveiling substantial interactions. These included hydrophobic interactions with amino acid residues His34, Lys353, Arg393, Arg403, Tyr449, Tyr495, Phe497, and Tyr505. Conversely, no ionic interactions were detected, but hydrogen bonds formed with His34, Lys353, Arg403, and Gly496. Amino acid residues Asp30, His34, Ala386, Arg393, Asp405, Glu406, Tyr453, and Ser494 were involved in water bridges. Figure 8G offers a timeline representation of these interactions with amino acid residues throughout the 150 ns simulation period, providing valuable insights into the dynamic behavior of the 3a_6MOJ complex at the molecular level. Figure 8H depict a 'ligand–protein' contact plot, highlighting interactions that persisted for more than 5.0% of the simulation time within the selected trajectory, spanning from 0.00 to 150.00 ns.

Several key amino acid residues emerged as central to these interactions. Arg403 exhibited hydrogen bond interactions for 49% of the simulation time, while Arg393 engaged in pi-cationic interactions for 38% of the time. Lys353 formed hydrogen bonds for 79% of the simulation duration, Gly496 participated in hydrogen bonds for 13%, and His34 engaged in hydrogen bonding interactions for a substantial 98% of the simulation period. Moreover, Fig. 7h, the 'ligand torsions plot,' summarizes the conformational changes observed within each rotatable bond (RB) in the ligand across the entire simulation trajectory from 0.00 to 150.00 ns. In summary, the findings unequivocally affirm the enduringly stable conformation of the ligand–protein complex throughout the 150 ns simulation period, underlining the significance of the identified key amino acid residues and highlighting the conformational changes within the ligand.

## Materials and methods

**Figure 9:**
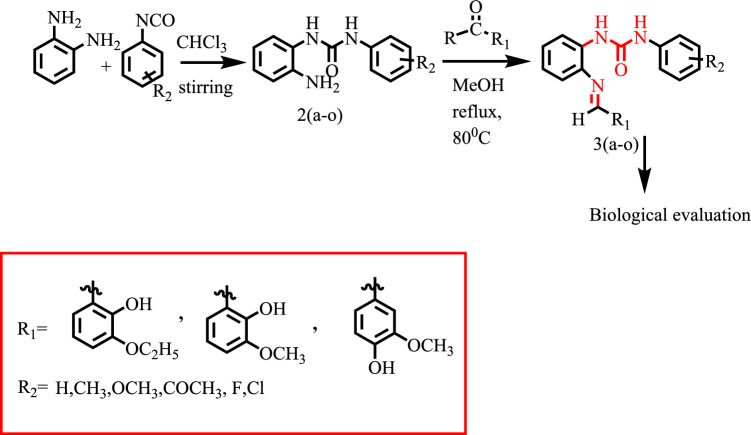
Synthesis of Schiff base 1,3-dipheny urea derivatives.

Without purification, all of the starting ingredients utilized in the synthesis were purchased from Sigma-Aldrich Co. (Germany). Other solvents, including methanol, pure ethanol, and others, were also acquired from various commercial sources in sufficient purity and utilized directly in the reaction media. Thin layer chromatography (TLC) was used using silica gel 60 aluminum-backed plates and the appropriate solvent system to monitor the reaction. Spotson TLC plates were seen by using UV light at a 254 nm wavelength. The infrared (IR) spectra were captured using a Shimadzu IR Affinity-I spectrophotometer between 400 and 4000 cm^−1^. DMSO-d6 and CDCl3 were used as solvents for the 1H and 13C nuclear magnetic resonance (NMR) spectra, which were then recorded at 25 °C using a Bruker spectrophotometer in a diluted solution at 300, 400, and 600 MHz. Coupling constants (J) were expressed in Hertz and chemical shifts were reported in parts per million (δ = ppm) (Hz). Singlet (s), doublet (d), triplet (t) and multiplet signals were used to define the signals (m). The Bruker Daltonics mass spectrometer was then used to record mass spectra (ESI–MS). The Stuart melting point device was used to determine the uncorrected melting points of cover slips. The urea derivatives were synthesized as depicted in Fig. 9.

### In vitro inhibition assay of SARS-CoV-2 spike protein

**Figure 10:**
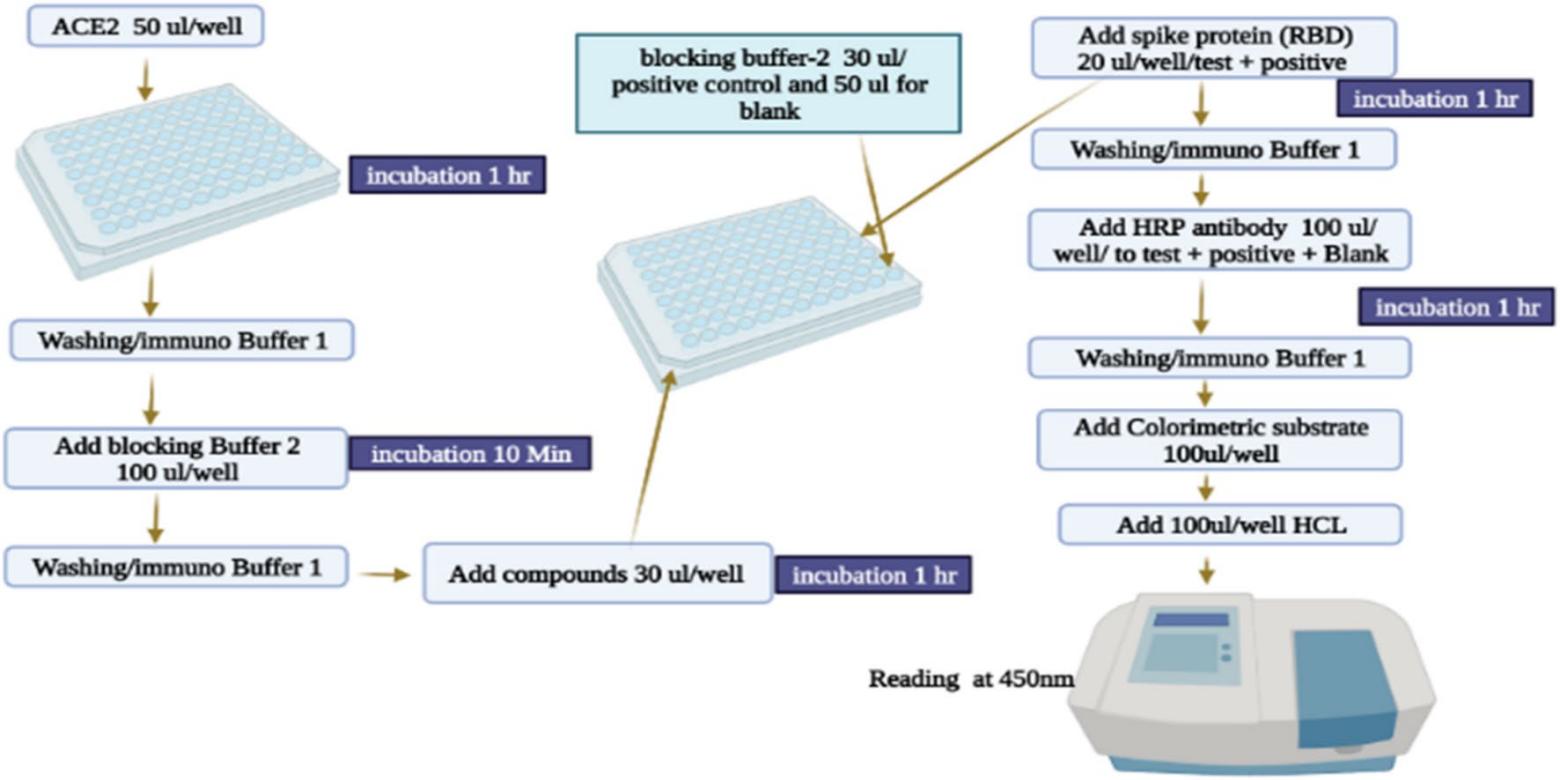
A step-by-step workflow diagram of the SARS-CoV-2 spike protein inhibition assay.

The binding of viral Spike proteins RBD to the hACE2 cell surface was assessed using an assay using the BPS Bioscience/Tebu-bio kit, situated in Offenbach, Germany^45^. The spike S1 RBD, Mouse Fc-fusion (SARS-CoV-2) was evaluated using a colorimetric-based assay to find chemical compounds that might be used to treat this severe infection^46^. A 96-well plate's bottom was coated with ACE2, at a concentration of 50 ng/well. After that, 100 ng/well of biotin-labelled RBD/S1 protein was added to enable for simple interactions with the ACE2 coating^47^. The ability of the complex S-protein to bind to ACE2 was examined using the streptavidin–horseradish peroxidase (HRP) substrate. The assay was carried out by adding 50 µL/well of ACE2 to a 96-well plate, which was gently shaken for one h at a particular temperature (25 °C). Using 100 µL/well washing buffer (1 × immune), the plate was cleaned and washed three times after incubation. Then 100 µL of blocking buffer was added into each well and followed by 10 min incubation, washed with washing buffer 1. Each substance was diluted using 70% ethanol, and screened for the concentration 0.5 mM, 30 µL/well. Then the 96-well plate was incubated for one h at room temperature with 30 µL/well-blocking buffer 2 as a positive control and 50 µL/well-blocking buffer 2 as a blank. The test wells and the positive control wells were added 20 L/well spike S1 (0.0625 ng/L) by following incubation. After incubation, 100 µL/well of HRP-labelled antibody 1 (anti-mouse), dissolved in blocking solution 2, was applied to the 96-well plate and incubated with gentle shaking at room temperature. Finally, the colorimetric HRP substrate, 100 L/well, was used and incubated at room temperature until the positive control's blue color appeared. Following the formation of the blue color, 100 µL/well of 1N HCl was added to each well, and measurements at 450 nm were taken. The % inhibition for each tested samples was calculated by using the following formula [Eq. (1)]. The flow chart of the in vitro activity is reported in Fig. 10.

1 $$\begin{array}{c}\%Inhibition=100-\left(\frac{{O.D}_{test compound}}{{O.D}_{control}}\right)\times 100\end{array}$$

### Selection of protein structure for molecular docking of compounds

For docking analysis, the spike protein crystal structure was obtained from protein data bank (RCSB) with PDB ID: 6MOJ^48^. The X-ray diffraction structure of the SARS-CoV-2 spike protein receptor binding domain (RBD) bound to the hACE2 receptor was selected because of its good resolution 2.45 Å. Amber14: EHT force field^49^ was utilized to clean unclear interactions and missing atoms in the protein crystal structure using MOE version (2022.09)^50^. MOE's Loop builder tool was used to simulate the protein chain's absent links. To better define where the protein begins and ends, charges were added to its terminals (C–N)^51^. Hydrogen bonds, partial charges, and Vander Wal forces were not present in the RBD of the spike protein until the Quick Prep module of the MOE was utilized^52^. That protein complex has had all its water molecules removed.

### Molecular docking analysis

Using the MOE Dock program, we were able to successfully dock the compounds at the RBD of spike protein. The hACE2 receptor's protein–protein interaction with the Spike protein RBD was chosen as the docking site for drugs. During docking, Triangle Matcher placement algorithm^53^ with rotated bonds was used to position each ligand at the RBD interface. Each docked pose of compounds were scored by chosen using the London dG^54^ scoring function and the 100 poses of each ligand were generated, later GBVI-WSA dg scoring system was used to choose the final 30 docked postures for each compound^55^. Protein–Ligand Interaction Fingerprints (PLIF) of the MOE were used to quantitatively determine the interactions and binding orientations of molecules at the RBD interface^56^. H-bonds, water-ligand-residue bridges, surface interaction, metal ligation, and arene attraction are just some of the protein–ligand interactions that can be estimated using PLIF.

### In-silico ADME calculations and BOILED-egg model analysis

To commence ADME calculations, we saved all ligand structures in the '.mol2' format and conducted necessary preparations using the 'LigPrep' module, developed by Schrodinger, Inc. in New York, USA, 2023. These computations employed the OPLS-2005 force field. The calculations were executed on a Lenovo ThinkPad® Workstation, equipped with a Ryzen 5 (5600 U) processor and 16 GB of memory, ensuring robust and efficient processing. Following this, we generated a comprehensive set of ADME (Absorption, Distribution, Metabolism, and Excretion) descriptors by employing the QikProp module, also developed by Schrodinger, Inc. in 2023, in normal mode. For the 'BOILED-egg' model analysis, we utilized the 'SwissADME' webserver, accessible at http://www.swissadme.ch/index.php, with the 'SMILES' strings of all compounds, allowing us to gather valuable insights into the properties of these compounds.

### Molecular dynamics simulation (MDS) study

To perform Molecular Dynamics Simulation (MDS) analysis, we conducted simulations on the complex labelled **'6MOJ_3a'** utilizing the 'Desmond' module, a software developed by Schrodinger, Inc., New York, USA, 2023. These simulations extended for a duration of 150 ns. In this simulation setup, we employed an explicit solvent model, which included TIP3P water molecules, and utilized the OPLS-2005 force field to represent the molecular interactions accurately. To ensure an accurate representation of the system, the simulation was conducted within a periodic boundary salvation box with dimensions measuring 10 Å × 10 Å × 10 Å. This choice of dimensions allowed us to simulate the behaviour of the complex in a controlled and realistic environment. To maintain a charge balance of 0.15 M in the system, sodium ions (Na^+^) were introduced. Additionally, NaCl solutions were incorporated to mimic physiological conditions, creating a more biologically relevant environment for simulation. The entire system, including the complex, solvent, and ions, was subjected to the MDS for a duration of 150 ns, providing insights into the dynamic behaviour of the **'6MOJ_3a'** complex under these specific conditions.

## Conclusion

Several viable antiviral strategies are available to stop virus entrance, replication, and dissemination within host cells including targeting the viral proteins and/or blocking cellular receptors. Hence, we designed the current study by considering the role of Schiff bases of 1,3-dipheny urea derivatives and their potential advantages against SARS-CoV-2 spike protein. In the preliminary investigation, we have identified twelve inhibitors with high inhibitory potency for spike protein. These substances can be used as a therapeutic approach during treatment for COVID-19 patients. Among the tested hits, eleven compounds including **3a** (96.00%), **3d** (89.60%), **3e** (84.30%), **3f** (86.20%), **3g** (88.30%), **3h** (86.80%), **3k** (82.10%), **3l** (90.10%, **3m** (93.49%), **3n** (85.64%), and **3o** (81.79%) inhibit SARS-CoV-2 spike protein in the range of > 80%. These new inhibitors of SARS-CoV-2 spike protein pave the way of developing these molecules as leads to combat SARS-CoV-2 infection.

### Supplementary Information

Supplementary Information.

## Data Availability

All data generated or analyzed during this study are included in this published article [and its Supplementary information files].
