# Supplementary material for: Structural, dynamic behaviour, in-vitro and computational investigations of Schiff’s bases of 1,3-diphenyl urea derivatives against SARS-CoV-2 spike protein
Source: Sci Rep. 2024 Jun 1;14:12588. doi: 10.1038/s41598-024-63345-9 (PMC11143201; doi:10.1038/s41598-024-63345-9)
Supplement: Supplementary file 1 — Supplementary Information. [file 41598_2024_63345_MOESM1_ESM.docx]

## Experimental

### General procedure for the synthesis of Schiff base 1,3-dipheny urea derivatives

Ortho phenylenediamine (**1**) (5mmol) was dissolved by steady stirring at room temperature in 15-20 ml of chloroform. Then, equimolar amounts of several substituted isocyanates were carefully dropped into this diamine solution using a dropping funnel. Immediately after stirring, a solid product precipitated out and was filtered before being washed with n-hexane and being vacuum-dried. The final products (**3a**-**3o**) were filtered, washed with cold ethanol, and dried under vacuum from the resultant mono substituted 1,3-diphenylurea’s (**2a**-**2o**) (1mmol) and substituted aldehydes (1mmol) refluxed in 8–10 mL of methanol. The above mentioned procedure of synthesis was followed from previous reported article.[1]

## Chemistry

Ortho phenylenediamine (**1**) was reacted with an equimolar amount of various substituted isocyanates under stirring conditions at room temperature, and the resulting mono substituted 1,3-diphenylureas (**2a**–**o**) were then refluxed with substituted aldehydes by simple condensation while being refluxed in methanol to obtain the final products (**3a**-**3o**)*.* By combining a variety of aldehydes, such as *o*-vanilline and 3-ethoxy salicylidehyde with various mono substituted 1,3-diphenylureas, the scope of the reaction was expanded. Good yields of the desired derivatives (**3a**–**3o**) were achieved (Scheme 1).

FTIR, ^1^H NMR, ^13^C NMR spectroscopy and Mass spectrometry were used to determine the structures of the Schiff base 1,3-dipheny urea derivatives. In the FTIR, the C=N band was seen between 1567 and 1614 cm-1. The existence of phenolic OH was confirmed by the ^1^H NMR signal, which showed up in the *δ* 10–12 Ppm range. Other measured peaks, including HC=N, were consistent with the structure expected. Other aromatic and aliphatic protons' spectrum data were consistent with these anticipated chemical structures. The molecular ion peaks in ESI spectra were [M+H] +, which was exactly in line with the molecular weight of the produced molecules.

3a

1-(3-chlorophenyl)-3-(2-((3-ethoxy-2-hydroxybenzylidene)amino)phenyl)urea

Yellow solid; Yield: 56%, m.p: 219-221 ^o^C; IR ʋ max (cm^-1^): 3300 (NH), 1614 (C=N), 1650(C=O), ^1^H-NMR (600 MHz, DMSO-*d^6^*) δ ppm; 11.92 (1 H, s), 9.56 (1 H, s), 8.90 (1 H, s), 8.28 (1 H, s), 8.06 (1 H, d, *J* = 8.1 Hz), 7.74 (1 H, s), 7.39 (1 H, d, *J* = 7.9 Hz), 7.27 (4 H, dt, *J* = 24.7, 8.0 Hz), 7.20 – 7.07 (2 H, m), 7.02 (1 H, d, *J* = 8.0 Hz), 6.93 (1 H, t, *J* =7.9 Hz), 4.09 (2 H, q, *J* = 7.0 Hz), 1.36 (3 H, t, *J* = 7.1 Hz); ^13^C NMR ppm; (151 MHz, DMSO) 163.1, 152.2, 149.9, 147.0, 141.2, 139.2, 133.2, 132.8, 130.4, 127.0, 123.3, 123.2, 121.5, 120.5, 120.3, 118.9, 118.8, 117.5, 116.7, 116.6, 64.1, 14.7 ;C_22_H_20_ClN_3_O_3_ (409.12) m/z (%): 410.11[M+H]+ (100)

3b

1-(2-((3-ethoxy-2-hydroxybenzylidene)amino)phenyl)-3-(4-fluorophenyl)urea

Yellow solid; Yield: 53%, m.p: 218-220 ^o^C; IR ʋ max (cm^-1^): 3298 (NH), 1613 (C=N), 1651(C=O), ^1^H-NMR (600 MHz, DMSO-*d^6^*)δ ppm; 11.95 (1 H, s), 9.39 (1 H, s), 8.90 (1 H, d, *J* = 2.3 Hz), 8.21 (1 H, d, *J* = 2.4 Hz), 8.08 (1 H, d, *J* = 8.1 Hz), 7.51 – 7.43 (2 H, m), 7.39 (1 H, d, *J* = 7.9 Hz), 7.25 (2 H, q, *J* = 8.1 Hz), 7.18 – 7.05 (4 H, m), 6.92 (1 H, td, *J* = 7.8, 2.3 Hz), 4.25 – 3.89 (2 H, m), 1.36 (3 H, t, *J* =5.9 Hz); ^13^C NMR δ ppm (151 MHz, DMSO) 163.09, 158.15, 156.5, 152.4, 149.9, 147.0, 138.9, 136.0, 133.1, 127.0, 123.3, 122.8, 120.3, 120.2, 119.9, 119.9, 118.8, 118.8, 116.7, 115.4, 115.2, 64.0, 14.7;C_22_H_20_FN_3_O_3_ (393.42) m/z (%): 394.13 [M+H]+ (100)

3c

1-(2-((3-ethoxy-2-hydroxybenzylidene)amino)phenyl)-3-(3-fluorophenyl)urea

Yellow solid; Yield: 56%, m.p: 220-222 ^o^C; IR ʋ max (cm^-1^): 2983 (NH), 1592 (C=N), 1651(C=O), ^1^H-NMR (600 MHz, DMSO-*d^6^*)δ ppm; 11.94 (1 H, s), 9.57 (1 H, s), 8.90 (1 H, s), 8.28 (1 H, s), 8.06 (1 H, d, *J* =8.1 Hz), 7.51 (1 H, d, *J* =11.9 Hz), 7.39 (1 H, d, *J* =7.9 Hz), 7.28 (3 H, td, *J* = 16.4, 15.7, 7.8 Hz), 7.16 (1 H, d, *J* =8.0 Hz), 7.10 (2 H, d, *J* =8.1 Hz), 6.93 (1 H, t, *J* =7.9 Hz), 6.78 (1 H, t, *J* =8.6 Hz), 4.09 (2 H, q, *J* = 6.7 Hz), 1.36 (3 H, t, *J* = 7.1 Hz); ^13^C NMR δ ppm; (151 MHz, DMSO) 163.2, 163.1, 161.6, 152.2, 149.9, 147.0, 141.6, 141.5, 139.1, 132.8, 130.4, 130.3, 127.0, 123.3, 123.2, 120.5, 120.3, 118.9, 118.8, 116.7, 113.9, 113.8, 108.2, 108.1, 104.9, 104.8, 64.1, 14.7 ;C_22_H_20_FN_3_O_3_ (393.42) m/z (%): 394.13 [M+H]+ (100)

3d

1-(2-((3-ethoxy-2-hydroxybenzylidene)amino)phenyl)-3-(naphthalen-2-yl)urea

Yellow solid; Yield: 61%, m.p: 229-231 ^o^C; IR ʋ max (cm^-1^): 2972 (NH), 1555 (C=N), 1651(C=O), ^1^H-NMR (600 MHz, DMSO-*d^6^*)δ ppm; 9.33 (1 H, s), 8.94 (1 H, d, *J* = 2.2 Hz), 8.63 (1 H, s), 8.15 (1 H, d, *J* = 8.4 Hz), 8.07 (1 H, d, *J* = 8.2 Hz), 7.92 (2 H, dd, *J* =13.7, 7.8 Hz), 7.67 (1 H, d, *J* = 8.3 Hz), 7.56 (3 H, dt, *J* = 23.6, 8.0 Hz), 7.48 (1 H, td, *J* = 8.0, 2.1 Hz), 7.37 (1 H, d, *J* = 7.9 Hz), 7.32 (1 H, d, *J* = 7.9 Hz), 7.27 (1 H, t, *J* = 7.8 Hz), 7.19 – 7.08 (2 H, m), 6.93 (1 H, td, *J* = 7.9, 2.2 Hz); ^13^C NMR δ ppm (151 MHz, DMSO) 163.2, 153.0, 150.1, 147.0, 139.2, 134.1, 133.7, 133.2, 128.3, 127.0, 126.5, 125.9, 125.8, 125.6, 123.5, 123.3, 123.1, 121.8, 121.0, 120.2, 118.9, 118.8, 118.7, 116.8, 64.1, 14.7 ;C_26_H_23_N_3_O_3_ (425.49) m/z (%): 426.17 [M+H]+ (100)

3e

1-(2-((3-ethoxy-2-hydroxybenzylidene)amino)phenyl)-3-(4-methoxyphenyl)urea

Orange Yellow solid; Yield: 58%, m.p: 203-205^0^C; IR ʋ max (cm-1): 2971 (NH), 1613 (C=N), 1645(C=O), ^1^H-NMR (600 MHz, DMSO-*d^6^*)δ ppm; 11.94 (1 H, s), 9.18 (1 H, s), 8.89 (1 H, d, *J* = 2.3 Hz), 8.13 (1 H, s), 8.09 (1 H, d, *J* = 8.2 Hz), 7.37 (3 H, td, *J* = 9.1, 4.4 Hz), 7.24 (2 H, q, *J* = 7.7 Hz), 7.15 (1 H, d, *J* =8.0 Hz), 7.06 (1 H, t, *J* = 7.7 Hz), 6.92 (1 H, td, *J* = 7.9, 2.2 Hz), 6.90 – 6.83 (2 H, m), 4.09 (2 H, q, *J* = 7.0 Hz), 3.71 (3 H, s), 1.36 (3 H, t, *J* = 7.0 Hz); ^13^C NMR δ ppm (151 MHz, DMSO) 163.0, 154.5, 152.5, 149.9, 147.0, 138.7, 133.3, 132.6, 127.0, 123.3, 122.6, 120.3, 120.0, 118.8, 118.8, 116.7, 114.0, 64.1, 55.1, 14.7 ;C_23_H_23_N_3_O_4_ (405.45) m/z (%): 406.16 [M+H]+ (100)

3f

1-(4-chlorophenyl)-3-(2-((3-ethoxy-2-hydroxybenzylidene)amino)phenyl)urea

Yellow solid; Yield: 62%, m.p: 219-221 ^0^C; IR ʋ max (cm^-1^): 2981 (NH), 1615 (C=N), 1650(C=O), ^1^H-NMR (600 MHz, DMSO-*d^6^*)δ ppm δ ppm; 11.93 (1 H, s), 9.49 (1 H, s), 8.89 (1 H, s), 8.25 (1 H, s), 8.07 (1 H, d, *J* = 8.2 Hz), 7.52 – 7.44 (2 H, m), 7.38 (1 H, d, *J* = 7.9 Hz), 7.34 – 7.29 (2 H, m), 7.25 (2 H, q, *J* = 8.1 Hz), 7.14 (1 H, d, *J* = 8.0 Hz), 7.08 (1 H, t, *J* = 7.7 Hz), 6.91 (1 H, t, *J* = 8.0 Hz), 4.08 (2 H, q, *J* = 7.1 Hz), 1.35 (3 H, t, *J* = 7.1 Hz); ^13^C NMR δ ppm (151 MHz, DMSO) 163.1, 152.2, 149.9, 147.0, 139.0, 138.6, 132.9, 128.6, 127.0, 125.4, 123.3, 123.0, 120.3, 120.3, 119.7, 118.8, 118.8, 116.7, 64.1, 14.7 ;C_22_H_20_N_3_O_3_ (409.12) m/z (%): 410.11[M+H]+ (100)

3g

1-(2-((3-ethoxy-2-hydroxybenzylidene)amino)phenyl)-3-(o-tolyl)urea

Orange Yellow solid; Yield: 66%, m.p: 217-219^0^C; IR ʋ max (cm^-1^): 3300 (NH), 1615 (C=N), 1648(C=O), ^1^H-NMR (600 MHz, DMSO-*d^6^*)δ ppm; 11.95 (1 H, s), 9.29 (1 H, s), 8.90 (1 H, d, *J* = 2.4 Hz), 8.21 (1 H, d, *J* = 2.4 Hz), 8.10 (1 H, d, *J* = 8.1 Hz), 7.39 (1 H, d, *J* = 7.9 Hz), 7.32 (1 H, s), 7.25 (3 H, dd, *J* = 14.7, 7.8 Hz), 7.16 (2 H, t, *J* = 7.9 Hz), 7.08 (1 H, t, *J* = 7.7 Hz), 6.93 (1 H, td, *J* = 8.0, 2.3 Hz), 6.79 (1 H, d, *J* = 7.5 Hz), 4.09 (2 H, q, *J* = 6.5 Hz), 2.28 (3 H, s), 1.36 (3 H, t, *J* = 6.0 Hz); ^13^C NMR δ ppm (151 MHz, DMSO) 163.0, 152.3, 149.9, 147.0, 139.6, 138.8, 137.9, 133.2, 128.6, 126.9, 123.3, 122.7, 122.6, 120.3, 120.2, 118.8, 118.8, 118.7, 116.7, 115.4, 64.0, 21.2, 14.7;C_23_H_23_N_3_O_3_ (389.46) m/z (%): 390.17 [M+H]+ (100)

3h

1-(2-((3-ethoxy-2-hydroxybenzylidene)amino)phenyl)-3-(m-tolyl)urea

Cream Yellow solid; Yield: 52%, m.p: 204-206^0^C; IR ʋ max (cm^-1^):3300 (NH), 1615 (C=N), 1650(C=O), ^1^H-NMR (600 MHz, DMSO-*d^6^*)δ ppm; δ H (600 MHz, DMSO-d6) 11.95 (1 H, s), 9.26 (1 H, s), 8.89 (1 H, d, *J* =2.2 Hz), 8.18 (1 H, d, *J* = 2.2 Hz), 8.10 (1 H, d, *J* = 8.1 Hz Hz), 7.39 (1 H, d, *J* =7.8), 7.35 (2 H, dd, *J* = 8.4, 2.2 Hz), 7.25 (2 H, q, *J* = 7.6 Hz), 7.15 (1 H, d, *J* = 8.0 Hz), 7.08 (3 H, t, *J* =7.7 Hz), 6.92 (1 H, td, *J* = 7.9, 2.1 Hz), 4.09 (2 H, q, *J* = 7.0 Hz), 2.24 (3 H, s), 1.36 (3 H, t, *J* = 5.9 Hz); ^13^C NMR δ ppm (151 MHz, DMSO) 163.0, 152.3, 149.9, 147.0, 138.8, 137.0, 133.2, 130.7, 129.2, 126.9, 123.3, 122.6, 120.3, 120.1, 118.8, 118.3, 116.6, 64.0, 20.3, 14.7;C_23_H_23_N_3_O_3_ (389.46) m/z (%): 390.17 [M+H]+ (100)

3i

1-(2-((3-ethoxy-2-hydroxybenzylidene)amino)phenyl)-3-(p-tolyl)urea

Cream Yellow solid; Yield: 53%, m.p:211- 213^0^C; IR ʋ max (cm-1): 3300 (NH), 1614 (C=N), 1649(C=O), ^1^H-NMR (600 MHz, DMSO-*d^6^*)δ ppm; 11.95 (1 H, s), 9.26 (1 H, s), 8.89 (1 H, d, *J* = 2.2 Hz), 8.18 (1 H, d, *J* = 2.2 Hz), 8.10 (1 H, d, *J* = 8.1 Hz), 7.39 (1 H, d, *J* = 7.8 Hz), 7.35 (2 H, dd, *J* = 8.4, 2.2 Hz), 7.25 (2 H, q, *J* = 7.6 Hz), 7.15 (1 H, d, *J* = 8.0 Hz), 7.08 (3 H, t, *J* = 7.7 Hz), 6.92 (1 H, td, *J* = 7.9, 2.1 Hz), 4.09 (2 H, q, *J* = 7.1, 6.1 Hz), 2.24 (3 H, s), 1.36 (3 H, t, *J* = 7.0 Hz); ^13^C NMR δ ppm (151 MHz, DMSO) 163.0, 152.3, 149.9, 147.0, 138.8, 137.0, 133.2, 130.7, 129.2, 126.9, 123.3, 122.6, 120.3, 120.1, 118.8, 118.3, 118.2, 116.6, 64.0, 20.3, 14.7; C_23_H_23_N_3_O_3_ (389.46) m/z (%): 390.17 [M+H] + (100)

3j

1-(2-((3-ethoxy-2-hydroxybenzylidene)amino)phenyl)-3-phenylurea

Yellow solid; Yield: 56%, m.p: 201-203 0C; IR ʋ max (cm^-1^): 2981 (NH), 1615 (C=N), 1649(C=O), 1H-NMR (600 MHz, DMSO-d6)δ ppm δ ppm; 11.97 (1 H, s), 9.36 (1 H, s), 8.90 (1 H, s), 8.23 (1 H, s), 8.10 (1 H, dd, J = 8.2, 1.4 Hz), 7.50 – 7.43 (2 H, m), 7.39 (1 H, dd, J = 7.9, 1.5 Hz), 7.26 (4 H, dddd, *J* =15.7, 14.3, 7.5, 1.7 Hz), 7.15 (1 H, dd, *J* = 8.0, 1.5 Hz), 7.11 – 7.05 (1 H, m), 6.99 – 6.95 (1 H, m), 6.92 (1 H, t, *J* = 7.9 Hz), 4.09 (2 H, q, *J* = 7.0 Hz), 1.36 (3 H, t, *J* =7.0 Hz); ^13^C NMR δ ppm (151 MHz, DMSO) 163.1, 152.3, 149.9, 147.0, 139.6, 138.9, 133.1, 128.8, 127.0, 123.3, 122.8, 121.8, 120.3, 120.2, 118.8, 118.8, 118.2, 116.7, 64.0, 14 ;C_22_H_21_N_3_O_3_ (375.16) m/z (%): 376.11[M+H]+ (100)

3k

1-(4-acetylphenyl)-3-(2-((3-ethoxy-2-hydroxybenzylidene)amino)phenyl)urea

Yellow solid; Yield: 63%, m.p: 208-210^0^C; IR ʋ max (cm^-1^): 3298 (NH), 1615 (C=N), 1649(C=O), ^1^H-NMR (600 MHz, DMSO-*d^6^*)δ ppm; 11.91 (1 H, s), 9.76 (1 H, s), 8.90 (1 H, d, *J* = 2.4 Hz), 8.36 (1 H, s), 8.08 (1 H, d, *J* = 8.1 Hz), 7.90 (2 H, dd, *J* = 8.7, 2.6 Hz), 7.59 (2 H, dd, *J* = 8.8, 2.5 Hz), 7.39 (1 H, d, *J* = 7.8 Hz), 7.27 (2 H, q, *J* = 8.1 Hz), 7.18 – 7.09 (2 H, m), 6.92 (1 H, td, *J* = 8.0, 2.5 Hz), 4.08 (2 H, q, *J* = 7.3 Hz), 1.35 (3 H, t, *J* = 6.9 Hz); ^13^C NMR δ ppm (151 MHz, DMSO) 196.2, 163.1, 152.0, 149.9, 147.0, 144.3, 139.2, 132.7, 130.4, 129.6, 127.0, 123.3, 120.5, 120.3, 118.9, 118.8, 117.1, 116.7, 64.0, 26.3, 14.7 ;C_24_H_23_N_3_O_4_ (417.47) m/z (%): 418.16) [M+H]+ (100)

3l

1-(3-chlorophenyl)-3-(2-((2-hydroxy-3-methoxybenzylidene)amino)phenyl)urea

Orange Yellow solid; Yield: 55%, m.p: 209-211 ^o^C; IR ʋ max (cm^-1^): 3301 (NH), 1614 (C=N), 1650(C=O), ^1^H-NMR (600 MHz, DMSO-*d^6^*)δ ppm; 11.83 (1 H, s), 9.56 (1 H, s), 8.91 (1 H, s), 8.30 (1 H, s), 8.06 (1 H, d, *J* = 8.2 Hz), 7.74 (1 H, s), 7.43 (1 H, d, *J* = 7.9 Hz), 7.27 (4 H, ddt, *J* = 22.7, 15.0, 8.0 Hz), 7.17 (1 H, d, *J* = 8.0 Hz), 7.10 (1 H, t, *J* = 7.7 Hz), 7.02 (1 H, d, *J* = 8.0 Hz), 6.94 (1 H, t, *J* = 7.9 Hz), 3.84 (3 H, s); ^13^C NMR ppm (151 MHz, DMSO) 162.5, 152.2, 149.6, 148.0, 141.2, 139.2, 133.2, 132.9, 130.5, 127.0, 123.2, 122.9, 121.5, 120.4, 118.9, 118.8, 117.6, 116.6, 115.5, 55.9 ; C_21_H_18_ClN_3_O_3_ (395.84) m/z (%): 396.10[M+H] + (100)

3m

1-(4-fluorophenyl)-3-(2-((2-hydroxy-3-methoxybenzylidene)amino)phenyl)urea

Yellow solid; Yield: 55%, m.p: 118-200 ^o^C; IR ʋ max (cm^-1^): 3301(NH), 1613 (C=N), 1649(C=O), ^1^H-NMR (600 MHz, DMSO-*d^6^*)δ ppm; 11.84 (1 H, s), 9.38 (1 H, s), 8.90 (1 H, s), 8.22 (1 H, s), 8.08 (1 H, d, *J* = 8.2 Hz), 7.52 – 7.38 (3 H, m), 7.25 (2 H, q, *J* = 7.8 Hz), 7.20 – 7.07 (4 H, m), 6.94 (1 H, t, *J* = 7.9 Hz), 3.84 (3 H, s); ^13^C NMR ppm (151 MHz, DMSO) 162.9, 158.6, 157.0, 152.8, 150.1, 148.4, 139.4, 136.4, 133.6, 127.4, 123.4, 123.3, 120.8, 120.6, 120.4, 120.4, 119.3, 119.2, 115.9, 115.8, 115.7, 56.4;C_21_H_18_FN_3_O_3_ (379.39) m/z (%): 380.13[M+H]+ (100)

3n

1-(3-fluorophenyl)-3-(2-((2-hydroxy-3-methoxybenzylidene)amino)phenyl)urea

Yellow solid; Yield: 51%, m.p: 202-203 ^o^C; IR ʋ max (cm^-1^): 3301 (NH), 1614 (C=N), 1650(C=O), ^1^H-NMR (600 MHz, DMSO-*d^6^*)δ ppm; 11.85 (1 H, s), 9.58 (1 H, s), 8.91 (1 H, s), 8.31 (1 H, s), 8.08 (1 H, dd, *J* = 8.2, 1.6 Hz), 7.52 (1 H, dt, *J* = 12.0, 2.2 Hz), 7.43 (1 H, dd, *J* = 7.9, 1.6 Hz), 7.35 – 7.20 (3 H, m), 7.17 (1 H, dd, *J* = 8.1, 1.6 Hz), 7.10 (2 H, td, *J* =7.6, 1.6 Hz), 6.94 (1 H, t, *J* = 7.9 Hz), 6.79 (1 H, td, *J* = 8.5, 2.3 Hz), 3.84 (3 H, s); ^13^C NMR ppm (151 MHz, DMSO) 163.2, 162.6, 161.6, 152.2, 149.6, 148.0, 141.6, 141.5, 139.2, 132.9, 130.4, 130.3, 127.0, 123.1, 122.9, 120.4, 118.8, 118.8, 115.5, 113.9, 113.8, 108.2, 108.1, 104.9, 104.8, 55.9 ;C_21_H_18_FN_3_O_3_ (379.39) m/z (%): 380.13[M+H]+ (100)

3o

(E)-1-(2-((2-hydroxy-3-methoxybenzylidene)amino)phenyl)-3-(naphthalen-2-yl)urea

Orange Yellow solid; Yield: 78%, m.p: 215-217 ^o^C; IR ʋ max (cm^-1^): 3301 (NH), 1614 (C=N), 1650(C=O), ^1^H-NMR (600 MHz, DMSO-*d^6^*)δ ppm; 12.12 (1 H, s), 9.33 (1 H, s), 8.95 (1 H, s), 8.65 (1 H, s), 8.15 (1 H, d, *J* = 8.4 Hz), 8.09 (1 H, d, *J* = 8.2 Hz), 7.93 (2 H, t, *J* = 6.6 Hz), 7.67 (1 H, d, *J* = 8.2 Hz), 7.60 – 7.55 (2 H, m), 7.48 (1 H, t, *J* = 7.9 Hz), 7.39 (1 H, d, *J* = 7.9 Hz), 7.33 – 7.23 (2 H, m), 7.17 (1 H, d, *J* = 8.0 Hz), 7.11 (1 H, t, *J* = 7.6 Hz), 6.95 (1 H, t, *J* = 7.9 Hz), 3.84 (3 H, s); ^13^C NMR ppm (151 MHz, DMSO) 162.8, 153.0, 149.8, 147.9, 139.2, 134.2, 133.7, 133.2, 128.3, 127.0, 126.5, 125.9, 125.9, 125.8, 125.7, 125.6, 123.4, 123.1, 123.0, 121.8, 120.8, 120.3, 118.8, 118.7, 115.5, 55.9 ;C_25_H_21_N_3_O_3_ (411.46) m/z (%): 412.16[M+H]+ (100)

1-(3-chlorophenyl)-3-(2-((3-ethoxy-2-hydroxybenzylidene)amino)phenyl)urea 3a

1-(2-((3-ethoxy-2-hydroxybenzylidene)amino)phenyl)-3-(4-fluorophenyl)urea 3b

1-(2-((3-ethoxy-2-hydroxybenzylidene)amino)phenyl)-3-(3-fluorophenyl)urea 3c

1-(2-((3-ethoxy-2-hydroxybenzylidene)amino)phenyl)-3-(naphthalen-2-yl)urea 3d

1-(2-((3-ethoxy-2-hydroxybenzylidene)amino)phenyl)-3-(4-methoxyphenyl)urea 3e

1-(4-chlorophenyl)-3-(2-((3-ethoxy-2-hydroxybenzylidene)amino)phenyl)urea 3f

1-(2-((3-ethoxy-2-hydroxybenzylidene)amino)phenyl)-3-(o-tolyl)urea 3g

1-(2-((3-ethoxy-2-hydroxybenzylidene)amino)phenyl)-3-(m-tolyl)urea 3h

1-(2-((3-ethoxy-2-hydroxybenzylidene)amino)phenyl)-3-(p-tolyl)urea 3i

1-(2-((3-ethoxy-2-hydroxybenzylidene)amino)phenyl)-3-phenylurea 3j

1-(4-acetylphenyl)-3-(2-((3-ethoxy-2-hydroxybenzylidene)amino)phenyl)urea 3k

1-(3-chlorophenyl)-3-(2-((2-hydroxy-3-methoxybenzylidene)amino)phenyl)urea 3l

1-(4-fluorophenyl)-3-(2-((2-hydroxy-3-methoxybenzylidene)amino)phenyl)urea 3m

1-(3-fluorophenyl)-3-(2-((2-hydroxy-3-methoxybenzylidene)amino)phenyl)urea 3n

(E)-1-(2-((2-hydroxy-3-methoxybenzylidene)amino)phenyl)-3-(naphthalen-2-yl)urea 3o

1. Pasha, A.R., et al., *Synthesis of new diphenyl urea-clubbed imine analogs and its implications in diabetic management through in vitro and in silico approaches.* Scientific Reports, 2023. **13**(1): p. 1877.
